# Creation of transgenic mice susceptible to coronaviruses: a platform for studying viral pathogenesis and testing vaccines

**DOI:** 10.18699/VJGB-22-49

**Published:** 2022-07

**Authors:** N.R. Battulin, O.L. Serov

**Affiliations:** Institute of Cytology and Genetics of the Siberian Branch of the Russian Academy of Sciences, Novosibirsk, Russia Novosibirsk State University, Novosibirsk, Russia; Institute of Cytology and Genetics of the Siberian Branch of the Russian Academy of Sciences, Novosibirsk, Russia Novosibirsk State University, Novosibirsk, Russia

**Keywords:** coronaviruses CoVs, SARS-CoV, MERS-CoV, COVID-19, transgenesis, “humanization” of the mouse genome, CRISPR/Cas9 technology, коронавирусы CoVs, SARS-CoV, MERS-CoV, COVID-19, трансгенез, «гуманизация» генома мышей, технология CRISPR/Cas9

## Abstract

Over the past 20 years, coronaviruses have caused three epidemics: SARS-CoV, MERS-CoV, and SARS-CoV2, with the f irst two having a very high lethality of about 10 and 26 %, respectively. The last outbreak of coronavirus infection caused by SARS-CoV2 in 2019 in China has swept the entire planet and is still spreading. The source of these viruses in humans are animals: bats, Himalayan civets, and camels. The genomes of MERS-CoV, SARS-CoV and SARS-CoV2 are highly similar. It has been established that coronavirus infection (SARS-CoV and SARS-CoV2) occurs through the viral protein S interaction with the lung epithelium – angiotensin-converting enzyme receptor 2 (ACE2) – due to which the virus enters the cells. The most attractive model for studying the development of these diseases is a laboratory mouse, which, however, is resistant to coronavirus infection. The resistance is explained by the difference in the amino acid composition of mouse Ace2 and human ACE2 proteins. Therefore, to create mice susceptible to SARS- CoV and SARS-CoV2 coronaviruses, the human ACE2 gene is transferred into their genome. The exogenous DNA of the constructs is inserted into the recipient genome randomly and with a varying number of copies. Based on this technology, lines of transgenic mice susceptible to intranasal coronavirus infection have been created. In addition, the use of the technology of targeted genome modif ication using CRISPR/Cas9 made it possible to create lines of transgenic animals with the insertion of the human ACE2 gene under the control of the endogenous murine Ace2 gene promoter. This “humanization” of the Ace2 gene makes it possible to obtain animals susceptible to infection with coronaviruses. Thus, transgenic animals that simulate coronavirus infections and are potential platforms for testing vaccines have now been created

## Abstract (ru)

За последние 20 лет коронавирусы вызвали три эпидемии, SARS-CoV, MERS-CoV и SARS-CoV2, причем летальность первых двух была очень высокой: около 10 и 26 % соответственно. Последняя вспышка коронавирусной инфекции, вызванная SARS-CoV2 в 2019 г. в Китае, охватила всю планету, и она все еще продолжает распространяться. Источником этих вирусов у человека были животные: летучие мыши, гималайские циветы и верблюды. Геномы MERS-CoV, SARS-CoV и SARS-CoV2 имеют высокое сходство между собой. Установлено, что заражение коронавирусной инфекцией (SARS-CoV и SARS-CoV2) происходит посредством контакта вирусного белка S с рецептором легочного эпителия – ангиотензин-конвертирующим ферментом 2 (АСЕ2), благодаря чему вирус попадает в клетки. Наиболее привлекательной моделью для исследования особенностей развития этих заболеваний является лабораторная мышь, которая, однако, резистентна к коронавирусной инфекции. Резистентность объясняется различием аминокислотного состава белков Асе2 мыши и АСЕ2 человека. Поэтому при получении мышей, восприимчивых к коронавирусам SARS-CoV и SARS-CoV2, в их геном переносят ген АСЕ2 человека. Экзогенная ДНК конструкций встраивается в реципиентный геном случайным образом и с варьирующим числом копий. На основе этой технологии были получены линии трансгенных мышей, восприимчивых к интраназальной коронавирусной инфекции. Применение технологии адресной модификации геномов с помощью CRISPR/Cas9 позволило получить линии трансгенных животных с инсерцией гена АСЕ2 человека под контроль промотора эндогенного гена Асе2 мыши. Такая «гуманизация» гена Ace2 дает возможность получить животных, наиболее близко имитирующих коронавирусную инфекцию человека. Таким образом, к настоящему времени создана серия линий трансгенных мышей – животных, моделирующих коронавирусные инфекции человека и потенциально способных служить платформами для тестирования вакцин.

## Introduction

The viral infection that caused severe acute respiratory syndrome (SARS) was first recorded in December 2019 in Wuhan, China, and rapidly spread around the world on a pandemic scale (Li et al., 2020; Zhou P. et al., 2020; Zhu et al., 2020). Soon, at the end of January 2020, it was published that the causative agent of the disease is a new type of coronavirus isolated from the bronchoalveolar secretions of six patients and called 2019-nCov (Zhu et al., 2020). Later, on the recommendation of the WHO (World Health Organization), the disease caused by the new SARS-CoV-2 virus was called COVID-19. Almost simultaneously (in early February 2020), additional data on the novel coronavirus infection from seven patients, six of whom were seafood vendors in the Wuhan market, were published (Zhou F. et al., 2020). According to P. Zhou et al. (2020), the SARS-CoV-2 virus genome shares 85 % similarity with the bat coronavirus and 79.6 % similarity with the previously described human SARS-CoV.

Model animals are an important tool in the research of many human pathologies. However, the creation of adequate animal models of infectious diseases has specifics associated with the high rate of co-evolution of the host-parasite system, during which both participants gain many specific adaptations. An example of this is the tropism of infectious human viruses based on the specific interaction of viral proteins with cellular receptor proteins, which gives rise to infection. The absence of the specific binding of viral proteins and target cell proteins causes resistance to a specific viral infection in different species.

On the other hand, differences in the response of the immune system to a particular viral agent in humans and animals can also become an insurmountable obstacle to the use of animals as model objects. Despite these difficulties, many mouse models of human viral diseases (poliomyelitis, measles, hepatitis B and C) have been created, which makes it possible to study the fundamental aspects of the development of a particular disease, such as infection processes, the course of a viral disease, and the interaction of the virus and the immune system. Such models have proven to be highly demanded for preclinical trials of new vaccines and antivirals (Takaki et al., 2017).

This review is devoted to the creation of laboratory mice susceptible to the SARS-CoV-2 and SARS-CoV coronaviruses in order to create an experimental platform for studying both the coronavirus pathogenesis itself and testing pharmacological antiviral drugs and vaccines.

## Human and animals coronavirus infections

The group of coronaviruses (CoVs) is represented by large enveloped viruses, the genome of which consists of singlestranded RNA (Lai et al., 2007). Coronaviruses are members of the subfamily Coronavirinae, family Coronaviridae, order Nidovirales. CoVs are composed of four genera: alpha-, beta-, gamma-, and deltacoronaviruses (Woo et al., 2009a, b), all of which cause zoonotic infections in animals. In the past two decades, of the entire cohort of coronaviruses, two have become pathogenic to humans, causing SARS and Middle East respiratory syndrome (MERS). In the first case, the outbreak originated in Guangzhou province in China in December 2002 and then spread to five continents (Peiris et al., 2003). According to the WHO, the SARS-CoV epidemic affected 8437 people, 813 of whom died. MERS-CoV coronavirus infection outbreak originated in the Arabian Peninsula in 2012 (Zaki et al., 2012) and spread to the Middle East, England and South Korea. According to the WHO, the epidemic affected 1728 people, with 624 cases of MERS-CoV infection resulting in death. It has been established that animals are the source of human infection with SARS-CoV and MERS-CoV (more details below).

## The process of infection with coronaviruses in humans and animals

To enter human target cells, SARS-CoV and MERS-CoV use their “crown”, which is represented by many spike-shaped (S) proteins. It has been established that the SARS-CoV S protein interacts with angiotensin-converting enzyme 2 (ACE2; encoded by the ACE2 gene) as an entry receptor (Li et al., 2003; Ge et al., 2013). During viral infection, the trimeric S protein is cleaved into S1 and S2 subunits, after which they are recognized by human cell receptors (Belouzard et al., 2009). Further, the S1 subunit containing the receptor-binding domain binds directly to the peptidase domain of the ACE2 protein, while S2 is responsible for membrane fusion (Li et al., 2005).

Based on this knowledge, several independent groups of researchers have proposed that the new SARS-CoV-2 coronavirus uses the same mode of entry into human cells as previously described SARS-like viruses. To confirm this hypothesis, comparisons were made between the protein sequences of SARS-like viruses and the new coronavirus SARSCoV- 2, and a high level of similarity was revealed. Further, to establish binding sites, a crystallographic analysis of the complex between the S1 subunit of the coronavirus and the human ACE2 protein was performed. As a result, it was found that the ACE2 protein has five key amino acid sequences that are involved in the binding of the S1 subunit of the virus (Lan et al., 2020; Wan et al., 2020; Wang et al., 2020).

The ACE2 gene in humans is expressed in the lungs, arteries, heart, brain, and small intestine and is an important component of the renin-angiotensin-aldosterone system (Bader, 2013). Expression of ACE2 in the lungs is mainly limited to alveolar epithelial cells of the second type. During coronavirus infection, ACE2 interacts with the receptor-binding domain of the virus spike protein, which leads to endocytosis of viral particles and their internalization (Kuba et al., 2010). These events result in severe acute respiratory syndrome, lung tissue damage, and extensive inflammation (Imai et al., 2005).

It is important to note that the first stages of coronavirus infections caused by SARS-CoV, MERS-CoV and SARSCoV2 have significant similarities.

## Technologies for the creation of transgenic mice for modeling coronavirus infections

As noted above, the source of human coronavirus diseases was animals. In 2003, Chinese researchers found that bats were carriers of the SARS-CoV coronavirus in humans through an intermediary – Himalayan civets, whose meat is considered a delicacy in Chinese cuisine (Guan et al., 2003; Peiris et al., 2003). The basis for this conclusion was 99.8 % similarity of the SARS-CoV genome with the virus isolated from bats and Himalayan civets. Bats are also natural carriers of the MERS-CoV coronavirus, and the camel is an intermediate carrier of the virus to humans (van Boheemen et al., 2012; Reusken et al., 2013).

It is worth noting that there are several animal species susceptible to SARS-CoV infection: ferrets, Syrian hamsters, cats, and several primates: macaques, African green monkeys, and marmosets (Glass et al., 2003; Martina et al., 2003; Roberts et al., 2005; Subbarao, Roberts, 2006). It is assumed that other animals may be susceptible to the novel coronavirus SARSCoV- 2 (Wan et al., 2020). However, these infected animals show minimal signs of impairment and generally lack the clinical symptoms associated with human coronavirus infection

In connection with the above, the strategy for creating model animals is based on the technology of introducing the human ACE2 gene, the main receptor for coronaviruses, into their genome. Indeed, in one of the first works on the creation of transgenic mice susceptible to coronavirus infection, a pK18-hACE2 recombinant DNA construct was developed, including the 5′-promoter and the 1st intron (with a mutation in the 3′-splice of the acceptor) of the human CK18 gene (encodes cytokeratin-18), as well as the translational enhancer alfa of the alfalfa mosaic virus (total size 2.5 kb), human ACE2 cDNA and a 3′ sequence including exon 6, intron 6, exon 7 and polyA signal element of the human CK18 gene (McCray et al., 2007). According to the authors’ intention, all elements were present in the construct to ensure a high level of its expression in epithelial cells. A purified DNA fragment of 6.8 kb excised from pK18-hACE2 was injected into the pronuclei of hybrid (C57BL/6Jx SJL/J) zygotes to obtain transgenic animals.

In the experiment described above (McCray et al., 2007), three lines of transgenic mice were obtained from different founders. It should be noted that the chosen technology provides random insertion of the transgene into the recipient genome, with a different number of copies. According to the authors, the number of transgene copies in the lines varied from 4 to 10. The transgene expression was observed in various tissues of the obtained mice: lungs, small intestine, liver and kidney, and at a low level was noted in the brain.

After intranasal SARS-CoV infection of transgenic CK18- hACE2, animals of all three lines died after the 7th day after virus inoculation. Moreover, mice carrying more transgene copies died already on the 4th day after infection. It is important to note that weight loss was observed in all transgenic mouse strains. The high titer of the virus was determined in the lungs compared with the control and reached the highest level on the 2nd day after infection. These data suggest increased viral replication as a key factor in the development of severe disease in transgenic animals. Interestingly, despite the expression of human ACE2 in the small intestine, liver, and kidney, the presence of the virus was not found in them. Among three tested lines of transgenic mice, only in one virus was detected in the brain at a low level, although the level of transgene expression was at the background level.

Histological analysis of the lungs on the 2nd day of infection showed signs of vascularization and peribronchiolar inflammation, and then there was an expansion of the zone of the inflammatory process, cell infiltration and desquamation of the cell epithelium in two lines of transgenic mice. In general, the pattern of intranasal infection of transgenic CK18-hACE2 lines showed similarities with the development of acute respiratory syndrome in humans caused by SARSCoV infection, in other words, these animals can be used as model objects for studying the pathogenesis of coronavirus infection (McCray et al., 2007; Netland et al., 2008). More recently, CK18-hACE2 infection of mice with SARS-CoV-2 has shown similarities with clinical manifestations of human COVID-19 (Yinda et al., 2020).

Almost simultaneously, another group of researchers created transgenic mice expressing human ACE2 under the control of the constitutive CAG promoter (Tseng et al., 2007). The cDNA sequence of the human ACE2 gene was inserted into the expression vector pCAGGS/MCS, which contained in the 5′-sequence of the enhancer of the early promoter of the cytomegalovirus fused with the promoter of the chicken actin gene, and in the 3′-region splicing sites of the rabbit globin gene. The total size of the pCAGGS-ACE2 expression vector was 7750 bp. A DNA fragment of this cassette was injected into the pronuclei of C57BL/6J × C3H/HeJ hybrid zygotes. Among the F0 offspring born from experimental zygotes, five transgenic animals were identified, of which two founders, AC70 and AC63, gave rise to two lines. RT-PCR analysis showed the presence of human ACE2 transgene transcripts in the stomach, heart, muscles, brain, kidneys, lungs, and small intestine.

Infection of transgenic mice with SARS-CoV showed the following symptoms: permanent weight loss, shortness of breath, and uncontrolled motor activity. The death of animals was observed after the 3rd day of infection and ended in total lethality by the 8th day. The reproduction of the virus occurred mainly in the lung tissue, while in other samples: swabs from the oral cavity, blood, heart, spleen, kidney, urine or feces, the virus was not detected. Summing up, the authors (Tseng et al., 2007) concluded that the resulting transgenic mouse lines are susceptible to SARS-CoV infection and exhibit external signs similar to those of humans, including the lethal outcome of infected animals. According to the authors, such mice may be useful for studying the pathogenesis of SARS-CoV infection.

In 2007, a third article on the creation of transgenic mice susceptible to SARS-CoV infection appeared (Yang et al., 2007). This group of researchers used a construct that included the mouse Ace2 gene promoter fused to the human ACE2 gene. The DNA of this construct was injected into the zygotes of ICR mice and the birth of transgenic animals was observed. Human ACE2 expression was detected in the lungs, heart, kidneys, and small intestine. On the 3rd and 7th days after infection with SARS-CoV, virus replication was observed in the lungs as well as signs of lung damage: interstitial hyperemia and hemorrhages, monocytic and lymphocytic infiltration, a proliferation of the alveolar epithelium and its desquamation. Interestingly, much later (Bao et al., 2020), after intranasal SARS-CoV-2 infection of these transgenic mice, a moderate weight loss was observed in the first five days, but no deaths were recorded in any case. The target and site of replication of COVID-19 was lung tissue, which resulted in the development of signs of pneumonia. Thus, the transgenic mouse line created in 2007 (Yang et al., 2007) has become a convenient platform for studying the pathogenesis of the two coronaviruses SARS-CoV and SARS-CoV-2.

Using the classical technology of transgenesis – microinjection of DNA expression vectors into the pronuclei of zygotes, which are randomly inserted into the recipient genome (Smirnov et al., 2020) and, which results in the expression of the transgene varying in different founders. One such study worthy of attention is the generation of transgenic C3B6 mice carrying human ACE2 under the control of an HFH4 promoter specific for ciliated lung epithelial cells (Ostrowski et al., 2003; Menachery et al., 2016). Human ACE2 expression was found in the lungs, brain, liver, and kidneys of transgenic HFH4-hACE2 mice. Intranasal infection with SARS-CoV or one of its WIV1-CoV strains caused weight loss in the first days of infection and death of animals after the 6th day from the moment of infection (Menachery et al., 2016). It is important that vaccines were tested on these mice and their positive effect was observed against both types of coronavirus. Later, these transgenic mice were successfully used to test antiviral therapy against COVID-19 (Jiang et al., 2020).

It makes sense to dwell on the study of the Russian group of A.V. Deikin, which for the first time provides protective measures to the researchers themselves against being infected with coronaviruses from transgenic mice (Bruter et al., 2021). The authors created a cassette consisting of two main elements: the pKB1 vector and the hACE2 open reading frame. pKB1 ampicillin-resistant vector was designed for cloning of genes, the expression of which depends on Cre-recombination (Cre-recombinase is present in the prokaryotic genome, but absent in eukaryotes), and contains insulators and terminators (“protecting” the transgene from the influence of nearby sequences), CAG promoter and STOP cassette. In addition, the vector contains the IRES element of the encephalomyocarditis virus, the GFP reporter gene, and the polyA signal of the SV40 virus. It is important that the expression of the human ACE2 transgene is activated only after removal of the STOP cassette by Cre-recombinase.

The recombinant DNA cassette was microinjected into F1 hybrid mouse zygotes (CBA × C57BL/6). The resulting transgenic animals did not express either human ACE2 or the reporter gene. To activate the transgene, transgenic mice were crossed with B6 mice Cg-Ndor1Tg(UBC-cre/ERT2)1Ejb/1 J (abbreviated as Ubi-Cre) carrying the Cre recombinase gene under the control of the UBC promoter. In heterozygous ACE2-GFP and Cre-transgene mice, the transgene was activated with tamoxifen, which activates Cre-UBC, which, in turn, cuts out the STOP cassette and activates the expression of ACE2 and the reporter gene (Bruter et al., 2021). Thus, heterozygous mice become susceptible to coronavirus infection (Dolskiy et al., 2022).

Indeed, direct experiments demonstrated that the virus intranasally inoculated to transgenic mice SARS-CoV2 induced thickening of alveolar duct septa mediated by diffuse hyperplasia of alveolar type II epithelium. Also, the lung tissue underwent lymphocyte infiltration. It should be emphasized that erythrocyte aggregates were present in the lung tissue in abundance, which was indicative of clotting. In contrast to lung tissue samples, no aberrations were found in the histological examination of the brain except for abundant erythrocyte aggregates as a sign of clotting (Dolskiy et al., 2022). All experimental transgenic mice died on day 5 to 10 after the intranasal inoculation.

The COVID-19 pandemic has stimulated the search for new technologies for creating model animals – laboratory mice. The development of targeted modification of human and animal genomes using CRISPR/Cas9 technology has opened up the prospect of obtaining “humanized” animals, in the genome of which target endogenous genes can be replaced by homologous human genes. An example is the insertion of the human ACE2 cDNA into the coding sequence of the endogenous Ace2 gene in C57BL/6 mice using CRISPR/Cas9 technology (Sun et al., 2020). The cDNA of the human ACE2 gene was inserted into exon 2 of the mouse Ace2 gene in mouse zygotes. Such an insert inactivated the endogenous Ace2 gene, and to visualize human ACE2 expression, the fluorescent protein reporter gene tdTomato (red glow) was inserted at its 3′-end, together with the IRES site and the polyA sequence. Among mice born from experimental zygotes, transgenic animals with a target insertion of the human ACE2 gene were identified.

Such mice were susceptible to intranasal SARS-CoV-2 infection at a young and adult age; the virus affected the lungs, trachea and brain. With intranasal infection, interstitial pneumonia developed, similar in manifestations to that of a person infected with SARS-CoV2, but without a lethal effect. It is clear that such genetically modified mice are seen as an attractive model of human coronavirus infection and could potentially serve as a platform for vaccine trials and pharmacological drug testing.

An essentially similar approach to “humanization” of the mouse Ace2 gene by insertion of the human ACE2 cDNA, has been implemented in C57BL/6 and BALB/c mouse embryonic stem cell (ESC) lines using CRISPR/Cas9 technology (Liu et al., 2021). It is appropriate to recall that the used ESC lines are capable, after injection into the cavity of tetraploid blastocysts, to replace endogenous cells of the internal mass, as a result of which transgenic descendants developed from donor ESCs are born. Transgenic mice thus generated, named C57BL/6NAce2em2(hACE2-WPRE, pgk-puro)/CCLA and BALB/c-Ace2em1(hACE2-WPRE, pgk-puro)/CCLA, were susceptible to intranasal SARS-CoV2 infection, although they differed from those obtained by S.-H. Sun et al. (2020) by a number of traits from transgenic mice. Thus, to date, a number of lines of “humanized” mice that carry the human ACE2 transgene and are susceptible to coronavirus infection and potentially capable of modeling human coronavirus pathology have been created.

## Conclusion

To sum up, it should be noted that despite the variety of created transgenic mouse lines susceptible to coronavirus infection (see the Table), the most popular among researchers are CK18- hACE2 mice created by the group P.B. McCray et al. (2007). According to PubMed, from 2020 to 2022, 101 articles that used this line as a model animal for the study of pathogenesis and coronavirus infection were published. Nevertheless, the development of new models continues, since the source of supply of mice of the CK18-hACE2 line is the Jackson Laboratory (USA), which supplies them only for experiments, without the right to breed them in national animal facilities in other countries.

**Table 1.**
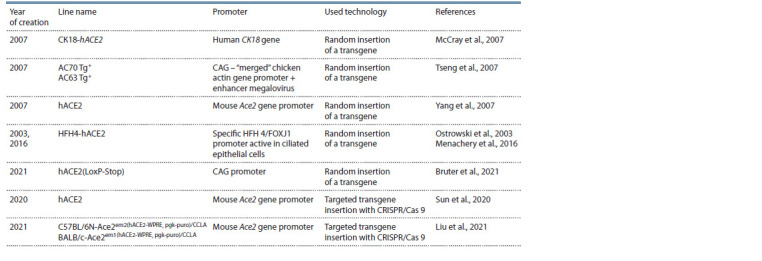
Transgenic mice with cDNA of the human ACE2 gene susceptible to SARS-CoV and SARS-CoV-2 coronavirus infection

## Conflict of interest

The authors declare no conflict of interest.
